# Ubiquitin Carboxyl-Terminal Hydrolase L1 and Its Role in Parkinson’s Disease

**DOI:** 10.3390/ijms25021303

**Published:** 2024-01-21

**Authors:** Olga Buneeva, Alexei Medvedev

**Affiliations:** Institute of Biomedical Chemistry, 10 Pogodinskaya Street, Moscow 119121, Russia; olbuneeva@gmail.com

**Keywords:** ubiquitin carboxyl-terminal hydrolase L1, ubiquitin–proteasome system, pathogenic mutations, Parkinson’s disease, experimental models

## Abstract

Ubiquitin carboxyl-terminal hydrolase L1 (UCHL1), also known as Parkinson’s disease protein 5, is a highly expressed protein in the brain. It plays an important role in the ubiquitin–proteasome system (UPS), where it acts as a deubiquitinase (DUB) enzyme. Being the smallest member of the UCH family of DUBs, it catalyzes the reaction of ubiquitin precursor processing and the cleavage of ubiquitinated protein remnants, thus maintaining the level of ubiquitin monomers in the brain cells. UCHL1 mutants, containing amino acid substitutions, influence catalytic activity and its aggregability. Some of them protect cells and transgenic mice in toxin-induced Parkinson’s disease (PD) models. Studies of putative protein partners of UCHL1 revealed about sixty individual proteins located in all major compartments of the cell: nucleus, cytoplasm, endoplasmic reticulum, plasma membrane, mitochondria, and peroxisomes. These include proteins related to the development of PD, such as alpha-synuclein, amyloid-beta precursor protein, ubiquitin-protein ligase parkin, and heat shock proteins. In the context of the catalytic paradigm, the importance of these interactions is not clear. However, there is increasing understanding that UCHL1 exhibits various effects in a catalytically independent manner through protein–protein interactions. Since this protein represents up to 5% of the soluble protein in the brain, PD-related changes in its structure will have profound effects on the proteomes/interactomes in which it is involved. Growing evidence is accumulating that the role of UCHL1 in PD is obviously determined by a balance of canonic catalytic activity and numerous activity-independent protein–protein interactions, which still need better characterization.

## 1. Introduction

Parkinson’s disease (PD) is a progressive neurodegenerative disorder characterized by a selective loss of dopaminergic neurons and the presence of Lewy bodies in the substantia nigra (SN) [1,2,3,4]. Although most cases of PD are sporadic, genes (and their protein products) associated with familial cases have been identified [5]. They are denominated as *PARK1, PARK2, PARK3*, etc. (see Table 1 for the top *PARK* gene-encoded proteins). Currently, more than 20 genes (and their protein products) associated with hereditary forms of PD are known [5,6,7,8], and the list of Park gene-encoded proteins has not been completed yet [9]. Although these inherited forms only account for less than 10% of all diagnosed cases of PD [10], the results of their studies have made an important contribution to our understanding of the mechanisms of the development of PD. Even a quick glance at Table 1 shows that most of them represent either components of the ubiquitin–proteasome system (UPS) or ubiquitination substrates.

**Figure 1:**
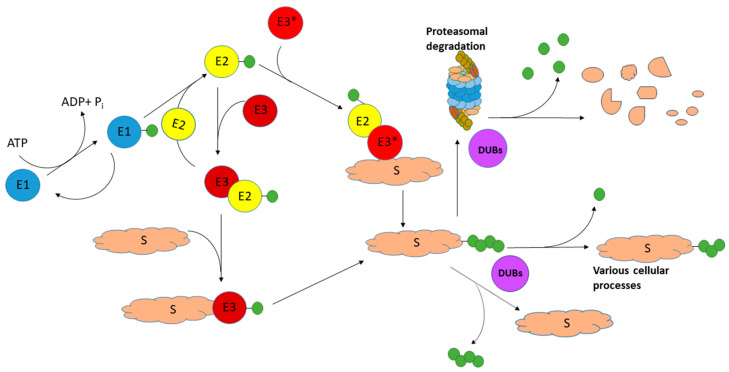
A simplified scheme illustrating the ubiquitination/deubiquitination process. The ubiquitination process includes several sequential stages that involve several enzymes: ubiquitin-activating enzyme (E1), ubiquitin-conjugating enzyme (E2), and ubiquitin ligase (E3). Their concerted action results in the covalent attachment of the ubiquitin moiety to target protein substrates (S) for proteasomal degradation. Deubiquitinases (DUBs) catalyze the cleavage of ubiquitin molecules from target proteins (and ubiquitinated protein remnants) and thus maintain (mono)ubiquitin levels in the cell for subsequent reuse. An asterisk at E3 shows the involvement of different ligases in the interaction with E2: RING E3 ligases (E3*) catalyze the transfer of ubiquitin directly from E2 to the substrate, while in the case of HECT or RBR E3 ligases (E3), the activated ubiquitin is first transferred from E2 to E3 and then transferred to the substrate from the E3 ligases. Ubiquitin molecules are shown as green circles (modified from [11]).

Ubiquitin ligases (E3) and deubiquitinases (DUBs) are essential enzymes of the UPS (Figure 1). One of their main functions is the maintenance of cell homeostasis, the timely production of new proteins, and the removal of damaged, misfolded, and aggregated proteins. Neurons are highly specialized cells with a long period of life; therefore, the proper functioning of UPS is especially important for the nervous tissue [33,34].

DUBs are proteases that hydrolyze the isopeptide bond between the lysine ε-amino group and the carboxyl group of the ubiquitin C-terminus [35,36].

To date, almost 100 different human DUBs are known [37,38]. These include the enzymes removing ubiquitin conjugates from PD-related proteins [34,39,40]. They constitute seven subfamilies. Most of them belong to the class of cysteine proteases (the only exception is the subfamily of JAB1/MPN/MOV34 metalloenzymes (JAMMs)). This unique group of DUBs, represented by metalloproteases, is characterized by the JAMM domain containing a catalytic zinc ion coordinated by two histidine residues, aspartate/glutamate, and a water molecule that is hydrogen-bonded to an adjacent glutamate [41]. Most JAMMs are highly specific for cleaving K63-linked polyubiquitin chains [36].

The biggest group of DUBs is represented by cysteine proteases. This group includes six subfamilies. Typical of them are the following: the most abundant subfamily of ubiquitin-specific proteases (USPs) [42,43], ovarian tumor domain proteases (OTUs) [44], some of which have a certain preference for hydrolysis of polyubiquitin chains with a few defined linkage types [36], ubiquitin C-terminal hydrolases (UCHs) [45,46], and Machado–Josephin domain-containing proteases (MJDs) [47]. The group of cysteine proteases also includes members of the motif interacting with ubiquitin-containing novel DUB family (MINDY), which are highly selective at cleaving K48-linked polyubiquitin chains [48,49], and newly discovered zinc finger-containing ubiquitin peptidases (ZUPs) [50,51], which are K63-specific and promote genome stability [52,53].

The catalytic mechanism of DUB cysteine proteases is very similar to that of papain. There are two or three key amino acid residues that make up the catalytic diad or triad. A Cys residue performs a nucleophilic attack on the isopeptide bond of ubiquitinated Lys. A nearby His side chain lowers the pKa of the Cys, facilitating a nucleophilic attack on isopeptide bonds. A third residue (Asn or Asp) aligns and polarizes the catalytic His. As this residue is not always essential for activity, some DUB cysteine proteases lack this residue and perform catalysis without it [35,36].

As it can be seen even from the names of some subfamilies, most of these DUBs are associated with cancer, and UCLH subfamily members are not the exception. Moreover, some of them are related to neuropathology [45,46]. However, only ubiquitin C-terminal hydrolase L1 (UCHL1) was classified as the Park5 protein.

Human ubiquitin carboxyl-terminal hydrolase L1 (UCHL1) was first discovered in the brain and other organs by two-dimensional electrophoresis and was termed PGP9.5 (protein gene product 9.5) [54]. Later, its ubiquitin C-terminal hydrolase activity was recognized [55].

In 1998, Leroy et al. identified a missense mutation in the fourth exon of the *UCHL1* gene in a German PD family. This mutation causing amino acid substitution (I93M), reducing the catalytic activity of this enzyme, was accompanied by increased protein aggregation [18]. This led to the inclusion of the *UCHL1* gene in the classification of PD-associated/PD-causative genes as *Park5* (Table 1) [56]. Interestingly, another mutation (S18Y, or third exon of *Park5*) reduced the risk of PD in some European [57,58], Chinese [59], and Japanese populations [60,61,62]. However, controversial results were obtained for populations in Italy [63], Australia [64], the United States [61], and afterwards for some populations in China [65,66] and Japan [67].

UCHL1 has been detected in the Lewy bodies of postmortem brains [68] and downregulated in patients with Lewy bodies [69]. Numerous experimental models using cell lines and animals provided good evidence for the role of UCHL1 in the development of PD.

Why does the deubiquitinase UCHL1 in particular play such an important role? In this review, we have tried to find an appropriate answer.

## 2. UCHL1 Is a Unique Member in the Family of UCHL Enzymes

UCHL1 belongs to the family of ubiquitin carboxy-terminal hydrolases (UCH), which also includes UCHL3, UCHL5, and BAP1 (BRCA1 (breast cancer early-onset 1)-associated protein 1) [46]. UCHL1, comprising 223 residues, is the smallest protein of this family, and its length has a significant impact on its catalytic properties. In vitro, UCHL1 (as well as UCHL3, comprising 231 residues) can hydrolyze various model substrates. These include ubiquitin (Ub)-ethyl ester (Ub-EtO), Ub conjugated with 7-amino-4-methylcoumarin (Ub-AMC), Ub derivatives, resembling *N^ε^*-ubiquitinated peptides similar to the degradation remnants that could be formed during proteasomal degradation of ubiquitinated proteins, Ub fused with decapeptides representing the first residues of UB itself, and also some other proteins (e.g., CEP52) [70,71,72]. In contrast to UCHL5 (329 residues), UCHL1 and UCHL3 cannot cleave the isopeptide bond of diUb chains [71,72] and therefore process large (poly) Ub chains [71,73]. Moreover, a several-fold increase (5X moles) of K-48-linked di-Ub or monoUb over the Ub-AMC caused inhibition of both UCHL1 and UCHL3 by 40–60% [74]. However, among the studied UCHs, only UCHL1 performed slow hydrolysis of the complete proubiquitin [71]. In the case of coexpression of UCHL1 and proubiquitin in *E. coli* cells, the efficiency of proubiquitin processing was high and represented more than 80% of the proubiquitin gene product [71]. The involvement of UCHL1 in the processing of the ubiquitin precursor obviously differs in various cells and depends on the expression level of this enzyme. For example, UCHL1 was not identified among deubiquitinases acting on mammalian ubiquitin precursors in HeLa cells [75]. Other authors did not find endogenous UCHL1 in HEK293 cells [76] and reported a very low level of this enzyme in HeLa cells [77].

These catalytic features of monomeric UCHL1 and other UCH (DUBs) are associated with the length of the so-called crossover loop [72,78], which is the shortest one in UCHL1 (11 residues). All the UCH DUBs do contain this unstructured loop, which restricts access to the active site [56]. In UCHL1, this crossover loop limits access to the active site to very short, disordered peptides (11 amino acids long) conjugated to Ub [78]. Genetic manipulations elongating the UCHL1 crossover loop by six residues resulted in the appearance of catalytic activity towards K48-K48-diUb (cleavage of the isopeptide bond) [72]. Interestingly, all UCHL1 mutants with the crossover loop longer than in UCHL3 (14 residues) were able to catalyze this reaction. This suggests that the length of the crossover loop determines catalytic activity and substrate specificity for isopeptide Ub chains [72].

In addition to the UCHL1 function as the DUB, a so-called E4 ligase function has been proposed for the UCHL1 dimer [79]. UCHL1 dimer formation has been detected at an enzyme concentration of 7 µM, and at 70 μM, UCH dimer became the predominant species. UCHL1 and α-synuclein coimmunoprecipitated from a rabbit brain synaptic vesicle fraction with a UCHL1 antibody [79]. The fact of UCHL1 coimmunoprecipitation with free and ubiquitinated alpha-synuclein supported the role of the UCHL1 dimer as a ubiquitin ligase [79]. However, subsequent studies revealed that UCHL1 did not exhibit ubiquitin ligase activity and did not ubiquitinate alpha-synuclein [80]. Moreover, existing knowledge cannot provide any rationale for polyubiquitin chain extension by UCHL1 due to substrate restriction access determined by the active site crossover loop [56]. These catalytic constraints should also be taken into consideration for the reappraisal of mechanisms underlying the role of UCHL1 in microtubule dynamics [77] and other effects, which are still considered in the context of direct ubiquitination of tubulins and microtubule-associated proteins via ligase activity [81,82,83,84].

Functional inhibition of UCHL1 by the specific reversible inhibitor LDN-5744 [85] or irreversible covalent modification/inhibition by methylmercury [86] caused a significant reduction of monoubiquitin in neuronal cells. Treatment of glioma U-87 MG cells with the UCHL1 inhibitor GSK13S also reduced the monoubiquitin level (but did not impair cell growth) [87].

Thus, in the current catalytic paradigm, UCHL1 should be considered the major hydrolase catalyzing proubiquitin processing and cleavage of ubiquitinated protein remnants, resulting in monoubiquitin formation. Binding of monoubiquitin by UCHL1 prevents its proteasomal degradation, thus maintaining a pool of free Ub within cells [88,89]. Other effects described in the literature are not associated with this catalytic activity, and understanding of this problem comes with the accumulation of new experimental data [89,90].

## 3. UCHL1 Is a Highly Expressed Protein That Interacts with Many Proteins in Brain Cells

UCHL1 is a small protein highly abundant in the brain, constituting from 1% to 5% of the soluble protein [55,91]. In the brain, it is mainly expressed in neurons; however, certain evidence now exists for its expression in oligodendroglial cells [92]. A recent postmortem study has shown that except for myelin basic protein (MBP), the level of UCHL1 is higher than the levels of other clinically important neuroglial proteins (glial fibrillary acidic protein, neurofilament light chain, tau) in most brain regions: pons, medulla oblongata, cerebellum, thalamus, and hippocampus [93]. In these regions, the average UCHL1 content ranged from 72 µg/g (cerebellum) to 190 µg/g (hippocampus) [93].

Although initially UCHL1 was considered a major protein of the cytosol of neurons [94,95] and was even named “neuron cytoplasmic protein 9.5” or “protein gene product 9.5 (PGP 9.5)”, up to 30–50% of this enzyme could be membrane-associated [55,79,96,97,98]. UCHL1 was membrane-associated in the primary cultured rat cortical neurons but not in the cultured cell lines of non-brain origin (HEK293T and COS7). Since UCHL1 lacks lipid-interaction domains, its association with membranes may occur indirectly via protein partners of macromolecular complexes [56,96].

The interaction of UCHL1 with macromolecular proteins is especially interesting in the context of UCHL1 identification among proteins associated with a fraction of rabbit proteasomes [99,100]. Proteomic profiling revealed the presence of UCHL1 in a fraction of rabbit brain proteasome (20S) core particles but not in 26S proteasomes [99,100]. This is consistent with the role of UCHL1 in the cleavage of ubiquitinated protein remnants to release and protect ubiquitin against proteolytic degradation by the proteasome core particle [101]. The association of UCHL1 with the rabbit brain 20S core particle was rather specific because it was not observed in rabbit liver 20S core particles and also in liver 26S particles [100].

Studies of putative protein partners of UCHL1 revealed about sixty individual proteins (Supplementary Table S1) located in all major compartments of the cell: nucleus, cytoplasm, endoplasmic reticulum, plasma membrane, mitochondria, and peroxisomes.

This implies UCHL1 localization in various subcellular compartments, which was demonstrated in various studies [56,81,96,102,103,104]. Table 2 lists some UCHL1-interacting brain proteins associated with the development of PD.

However, in the context of the current catalytic paradigm, defining this enzyme as a hydrolase of ubiquitinated protein remnants but not a ligase, a possible role of most UCHL1 interactions, given in Table 2 and Table S1, remains unclear. Interactions of UCHL1 with HSP90 and peroxiredoxin-2 are considered interactions between chaperones and a client protein aimed at protecting the enzyme involved in maintaining a free ubiquitin pool [115]. At the same time, it is really difficult to explain how the loss of UCHL1 rescues the defects related to PD by influencing pyruvate kinase M (PKM) in terms of the altered catalytic activity of UCHL1 [83]. PKM interacts with many proteins in the brain [123], and the effect of UCHL1 deficiency on the destabilization of PKM obviously involves changes in the PKM interactome rather than suppression of catalytic activity by UCHL1, which cannot act on such a large oligomer protein. It appears that besides catalytic functions, non-catalytic functions of UCHL1 also exist [89]. For example, known data about the localization of UCHL1 in mitochondria [88,103,107,108,109,110,111,112,116,117] and numerous changes in mitochondrial metabolism in response to manipulations with UCHL1 expression [124] support the idea about the non-catalytic functions of UCHL1 [89].

## 4. Mutations and Posttranslational Modification of UCHL1 Have a Significant Impact on the Catalytic Activity and Aggregability

Most spontaneously or artificially induced mutations are accompanied by a decrease in the catalytic activity of UCHL1. These include not only any residue of the catalytic trial (C90, H161, and D176) but also residues located at different sites of the polypeptide chain (see Table 3).

These mutations causing a reduction or abolishment of the hydrolase activity result in a decrease in the monoubiquitin level and an increase in the level of ubiquitinated proteins in the cell [88].

Some mutations had a significant impact on UCHL1 solubility. For example, C-terminal deletion of four residues increased membrane association and decreased solubility [96]. The N-terminal truncated mutant was also aggregation-prone [125]. The I93M mutation associated with familial PD not only decreased the hydrolase activity of UCHL1 but also reduced its solubility [111,112].

Proteomic analyses of PD brains revealed that five methionine residues (M1, M6, M12, M124, and M179) underwent oxidation to methionine sulfoxide, while the cysteine residue (C220) near the C-terminus was oxidized to cysteine sulfonic acid [130]. However, proteomic analysis of brain mitochondria from mice with MPTP-induced Parkinsonism did not reveal UCHL1 among oxidatively modified proteins [103]. It appears that such modification may occur during later stages of PD progression rather than after MPTP administration, causing acute movement disorder.

In vitro, 1-(3,4-dihydroxybenzyl)-1,2,3,4-tetrahydroisoquinoline, an endogenous parkinsonism-inducing dopamine derivative, binds to UCHL1 specifically at Cys152. Treatment of SH-SY5Y cells inhibited UCHL1 activity and decreased its solubility [131].

In vitro, oxidation of recombinant UCHL1 treated with 4-hydroxynonenal, a candidate endogenous mediator of oxidative stress-induced neuronal cell death, caused a loss of its hydrolase activity [126]. Nitration of purified UCHL1 with peroxynitrite resulted in nitration of Tyr80 [132]. The nitrosylated UCHL1 may be involved in the noncanonical transnitrosylation network, which transfers NO from UCHL1 to cyclin-dependent kinase 5 and then to dynamin-related protein 1; realization of such a scenario may cause serious consequences, including mitochondrial fragmentation and consequent synapse loss and cognitive impairments [133].

Covalent binding of cyclopentenone prostaglandin (CyPg) 15-deoxy-Δ12,14–prostaglandin J2 to UCHL1 Cys152 results in aggregation and/or disruption of UCHL1 enzyme activity [134]. Mutation of Cys152 (but not the five other cysteine residues) prevented the unfolding of this protein and preserved the UCHL1 hydrolase activity after incubation of the UCHL1 recombinant protein with CyPgs [134].

Thus, by analogy with other oxidized proteins exhibiting various functions in the cells, oxidized UCHL1 may influence and/or regulate many types of protein functions [90]. However, in contrast to many oxidized proteins, a high abundance of UCHL1 in the brain may cause much more powerful interventions in protein–protein interactions (and interactomes) in various brain cells.

## 5. Experimental Models of UCHL1 Deficiency and Postmortem Brain Studies Demonstrate a Role of This Protein in PD Pathogenesis

The gad (gracile axonal dystrophy) mice have a spontaneously developed autosomal recessive mutation caused by an in-frame deletion including exons 7 and 8 of the gene encoding UCHL1 [89,135,136]. The truncated UCHL1 variant lacks a segment of 42 amino acids containing a catalytic His161 residue, and the mice have typical neurodegenerative phenotypes: sensory and motor ataxia and the formation of inclusions in axon nerve terminals. They have a decreased level of monoubiquitin and form protein inclusions in vivo. However, such a typical symptom of PD as cell degeneration in SN was not observed in the gad mice [137]. In addition, the lack of wild-type (WT) UCHL1 insignificantly influenced the dopaminergic system, as evidenced by similar levels of striatal tyrosine hydroxylase (TH) and dopamine transporter (DAT) in gad and WT mice [138]. Treatment of gad and WT mice with the neurotoxin MPTP had statistically indistinguishable effects on the level of dopamine and its metabolites as well as on the number of TH-positive neurons in SN [138]. However, mouse nigral neurons, retrogradely transduced with S18Y UCHL1, but not WT UCHL1, were significantly protected against MPTP toxicity. Primary rat cortical neurons transduced with S18Y UCHL1 demonstrated a significantly higher survival rate than the cells transduced with WT UCHL1 [138]. These data indicate that the S18Y variant of UCHL1, but not WT UCHL1, exhibits neuroprotective activity in the cell and mouse models of Parkinson’s disease. Earlier, it was also demonstrated that overexpressed UCHL1^S18Y^ exhibited antioxidant functions in the culture of SH-SY5Y cells and mouse embryonic primary cortical neurons and did not influence proteasome functioning [139]. Since the catalytic activity of the UCHL1^S18Y^ mutant insignificantly differs from that of WT UCHL1 (see Table 3), it appears that the protective effect of UCHL1^S18Y^ should be obviously attributed to the non-catalytic functions of this protein.

Spontaneous mutation *nm3419*, which inserted a premature stop codon that truncated the last 78 amino acids of UCHL1 (and the loss of its catalytic activity), led to a 20% decrease in free monomeric ubiquitin level [140]. This was accompanied by up-regulation of lysosomal but not proteasomal components [140]. However, the total Ub-AMC hydrolyzing activity remained basically unchanged in hippocampal extracts of wild-type (+/+), nm3419 heterozygous (+/−), and nm3419 homozygous (-/-) mice. This suggests that other deubiquitinating enzymes can participate in this cleavage. Moreover, even more pronounced changes (both in terms of the monoubiquitin level and electrophysiology) have been found in mice with a loss of the proteasomal ubiquitin-specific protease Usp14 [140].

The catalytically inactive NT-UCHL1 lacking N-terminal 11 amino acids, which was stably expressed in HeLa cells, protected these cells against the PD-related toxin, rotenone, hydrogen peroxide, or the mitochondrial oxidative phosphorylation uncoupler, CCCP [125]. Interestingly, the levels of both transiently and stably expressed NT-UCHL1 in cells were lower than those of wild-type UCHL1. Despite the fact that NT-UCHL1 is more aggregation-prone, it exhibits neuroprotective activity, possibly due to its short half-life (3–6 h versus more than 24 h in UCHL1) and faster degradation than UCHL1 [125]. In the experimental model of MPTP-induced Parkinsonism, transgenic mice expressing human NT-UCHL1 had higher levels of tyrosine hydroxylase-positive cells in SN than in non-transgenic animals in the control group. This indicates the protective effect of the catalytically inactive UCHL1 on dopaminergic neurons.

UCHL1 knockout (KO) mice were characterized by total degeneration of presynaptic terminals at the neuromuscular junctions, impaired synaptic transmission, and also by progressive paralysis and premature death [141].

The familial PD-associated UCHL1 mutations replicated in animal and cell models confirm the role of UCHL1 in neuronal function and pathology. The UCHL1^I93M^ transgenic mice (I93M) reproduced the autosomal-dominant mutation in UCHL1 first identified in a German family with late-onset PD symptoms [18]. The UCHL^1I93M^ mice showed behavioral and physiological phenotypes of PD. At 20 weeks of age, high-expressing UCHL^1I93M^ mice had a significant reduction in the dopaminergic neurons in the SN and the dopamine content in the striatum. Although these changes were not observed in low-expressing UCHL^1I93M^ mice at this age, administration of MPTP caused a significant reduction in TH-positive dopaminergic neurons as compared to transgenic (Tg) mice expressing WT UCHL1 or non-Tg mice. Moreover, a slight increase in the insolubility of UCHL1 in the SN fraction from UCHL1^I93M^ transgenic mice was seen as compared to non-Tg mice [142].

Although postmortem studies of the brains of PD patients usually show hallmarks of the disease outcome, the most pronounced changes in the protein of interest (and its gene) provide valuable information about its possible role in this disease. The pilot study was performed using formalin-fixed, paraffin-processed sections known to contain ubiquitin-protein conjugate immunoreactivity in cortical Lewy bodies, neurofibrillary tangles, Rosenthal fibers, Pick bodies, and spinal inclusions in motor neuron disease [68]. The majority of cortical Lewy bodies in diffuse Lewy body disease showed immunoreactivity for PGP 9.5 (UCHL1) [68]. In the case of Alzheimer’s disease, only a minority of neurofibrillary tangles were immunostained, along with a minority of neurites surrounding senile plaques. There was clear differential detection of UCHL1 in different forms of ubiquitinated inclusion bodies in the nervous system [68]. UCHL1, especially its oxidized form, has been found in the ubiquitinated inclusion bodies of postmortem brains of patients with PD and Alzheimer’s disease [69,131,143] and patients with other neurodegenerative disorders (e.g., dementia with Lewy bodies, Alzheimer’s disease) [68,69,144]. The immunohistochemistry study of midbrain sections of a patient with sporadic PD showed alpha-synuclein- and UCHL1-double-positive Lewy bodies in SN DA neurons, suggesting physical and/or functional interaction between the two pathogenically important proteins in the human PD brain [145].

The UCHL1 mRNA [69] and the protein level of UCHL1 were downregulated in the brains of patients with idiopathic PD [69,130,143]. Three human brain UCHL1 isoforms have been identified with the proteomic study: the full-length form (a major target of oxidative damage) and two amino-terminally truncated forms. UCHL1 from PD brains was characterized by increased (up to 10-fold) levels of oxidized UCHL1 [130].

## 6. Mechanisms Underlying the Contribution of UCHL1 to the Pathogenesis of PD

Taking into consideration a decrease in the UCHL1 level in SN, the principal brain area affected by PD [146], which was observed in experiment models of MPTP-induced PD in monkeys [147] and mice [148] and in postmortem brain samples (see above), the mechanisms underlying the contribution of UCHL1 to the PD pathogenesis obviously include the following: (I) a decrease in the quantity of catalytically competent enzyme UCHL1; (II) changes in specific protein–protein interactions involving UCHL1; and (III) nonspecific changes associated with accumulation of the oxidatively modified abundant brain protein.

(I) A decrease in the quantity of catalytically competent UCHL1 was accompanied by reduced monoubiquitin levels needed for effective ubiquitination of client proteins [88]. In addition, wild-type UCHL1 but not the catalytically dead (C90S) mutant decreased ubiquitination of PKM and stabilized this key glycolytic enzyme [83]. Although the exact chain of molecular events remains unclear (UCHL1 cannot perform deubiquitination of large proteins; see Section 3), UCHL1 KO cells had an ATP level that was half lower than in WT UCHL1 cells [83].

(II) The genetic experiments, either in *Drosophila* or in mammalian cells, showed that loss of UCHL1 promoted mitophagy through a mitochondrial outer membrane protein known as the FUN14 domain-containing 1 (FUNDC1) protein [83]. This recently identified mitochondrial outer membrane protein induces receptor-mediated mitophagy [149]. UCHL1 physically interacts with several proteins involved in chaperone-mediated autophagy (CMA). The UCHL1^I93M^ mutant demonstrated a great enhancement of these interactions, which led to CMA impairment and the accumulation of alpha-synuclein [111,112]. The accumulation of alpha-synuclein and other PD-related proteins causes dysfunction in autophagy and mitochondrial clearance [150]. 

(III) Despite the fact that acute treatment of mice with MPTP was not accompanied by oxidative modification of brain mitochondrial UCHL1 [101], oxidized UCHL1 was detected in the postmortem brains of PD patients among other oxidized brain proteins forming Lewy bodies [130,145]. Since the nitrosylated UCHL1 may participate in the noncanonical transnitrosylation pathway [133], it is also possible that oxidized UCHL1 may be involved in the formation of pathological protein networks responsible for subsequent PD progression.

## 7. Conclusions

UCHL1 is a highly expressed protein in the brain, where it represents from 1% to 5% of cytosol protein. Its role in the development of PD is determined by several major issues: (a) UCHL1 is a unique enzyme responsible for ubiquitin precursor processing; (b) UCHL1 interacts with many important intracellular targets, including PD-related proteins; (c) mutations, posttranslational modifications of UCHL1, and interactions with PD-related proteins have a profound effect on cell functioning. However, a decrease or even loss of its catalytic activity accompanied by monoubiquitin deficiency did not necessarily result in neurodegeneration. Moreover, in contrast to SN, in brain areas resistant to MPTP action (arcuate nucleus), the UCHL1 content even increased in response to MPTP administration [148]. In this context, it is especially interesting that the N-terminal truncated mutant, lacking UCHL1 activity, effectively protected SN dopaminergic neurons in the experimental model of MPTP-induced PD [125]. A higher turnover rate of this UCHL1 mutant suggests that prevention of UCHL1 aggregation may be an important factor in the protection of PD development [125]. The increased aggregability of modified UCHL1 is consistent with the identification of UCHL1-binding proteins in the Lewy bodies of postmortem brains of PD patients (Table 2). Interacting with various intracellular proteins, UCHL1 plays a role in mitochondrial bioenergetics [124], regulation of glycolysis [73], chaperone-mediated autophagy [111], etc. However, these interactions cannot be explained in the context of known catalytic activity. Thus, there is growing evidence that the role of UCHL1 in PD is obviously determined by a balance of canonic catalytic activity and numerous activity-independent protein–protein interactions, which still need better characterization. It appears that the latter is a common feature of the Park-classified proteins. The DJ-1 protein, however, known as Parkinson’s disease protein 7, also performs catalytic and non-catalytic actions, which play different roles in PD development [151].

## Figures and Tables

**Table 1:** Some PARK-designated genes and their protein products involved in inherited forms of Parkinson’s disease (modified from [11]). The original table compiled from [12,13] was supplemented by [11].

Symbol	Gene	Protein Product	Relation to UPS	Type of PD	Inheritance	References
*PARK1*	*SNCA*	Alpha-synuclein	Ubiquitination substrate	Classical and early-onset PD	AD *	[14,15]
*PARK2*	*Parkin*	Parkin	E3 ubiquitin ligase	Early-onset PD	AR **	[16,17]
*PARK5*	*UCHL1*	Ubiquitin C-terminal hydrolase L1	Deubiquitinase	Classical PD	AD	[18]
*PARK6*	*PINK1*	PTEN-induced kinase 1	Phosphorylates ubiquitination substrate E3 ubiquitin ligase	Early-onset PD	AR	[19,20]
*PARK7*	*DJ-1*	DJ-1	Ubiquitination substrate	Early-onset PD	AR	[21,22,23]
*PARK8*	*LRRK2*	Leucine-rich repeat kinase 2	Ubiquitination substrate	Classical PD	AD	[24,25,26]
*PARK10*	* USP24 *	Ubiquitin-specific peptidase 24	Deubiquitinase	Late-onset PD	Risk factor	[27,28]
*PARK11*	*GIGYF2*	Grb10-interacting GYF protein-2	Could promote ligand-induced *ubiquitination* of IGF1R	Classical PD	AD	[29,30]
*PARK13*	*HTRA2*	Htra2 serine peptidase 2	Cell stress response	Susceptibility locus for PD	AD	[31]
*PARK15*	*FBXO7*	Fbxo7	E3 ubiquitin ligase	Early-onset PD	AR	[32]

* AD, autosomal dominant; ** AR, autosomal recessive.

**Table 2:** UCHL1-interacting proteins associated with PD development.

#	Accession Number	Gene Name	Protein Name	Function	Localization	References
1	P00533	EGFR	Epidermal growth factor receptor	3	PM, EPR, M	[105]
2	P05067	APP	Amyloid-beta precursor protein	3	PM, EPR, M	[106]
3	Q14568	HSP90 *	Heat Shock Protein 90-alpha A2	4	C, N, Mch, PM	[107,108,109,110,111,112]
4	P37840	SNCA *	Alpha-synuclein	3	C, M, N	[79,92,113]
5	P32119	PRDX2	Peroxiredoxin-2	4	C	[114,115]
6	P62987	UBA52 *	Monoubiquitin	5	C, N, PM, Mch	[88]
7	O60260	PARK2 *	E3 ubiquitin-protein ligase parkin	5	C, N, EPR, Mch	[116,117]
8	P34931	HS71L *	Heat shock 70 kDa protein 1-like	4	C, N	[111,112]
9	P14618	KPYM	Pyruvate kinase PKM	1	C	[83]
10	-	-	Tubulins ^a^	2	C	[77,92]

Localization: C—cytoplasm; EPR—endoplasmic reticulum; M—membranes; PM—plasma membrane; Mch—mitochondria; N—nucleus. Functional groups: 1. Proteins involved in energy generation and carbohydrate metabolism; 2. Proteins involved in cytoskeleton formation and exocytosis; 3. Protein involved in signal transduction and regulation of enzyme activity; 4. Antioxidant and protective proteins/enzymes; 5. Enzymes involved in the metabolism of proteins, amino acids, and other nitrogenous compounds. The full list of UCHL1-interacting proteins identified in various model studies and corresponding references are given in Supplementary Table S1. An asterisk marks the proteins identified as particular components of Lewy bodies [107,108,109,110,113,117,118,119,120,121,122]. ^a^ Particular tubulins have not been described in the cited study.

**Table 3:** Hydrolase activity of UCHL1 mutants.

Mutation	Hydrolase Activity Versus WT UCHL1, %	Reference
N-terminal deletion (residues 1–11)	0	[125]
E7A	50%	[80]
S18Y	113%	[126]
D30K	0	[88]
Q73R	97%	[88,127]
C90S	<<1% or 0	[88,127]
I93M	50%	[18]
C152A	~85%	[128]
H97Q, H97N	85–87%	[127]
H161D	<1%	[127]
H161K, H161N, H161Q, H161Y	<<1%	[127]
D176N	2.5%	[127]
R178Q	400%	[129]
A216N	Not measurable	[129]
C-terminal deletion (residues 220–223)	Not determined	[115]

## Supplementary Materials

The following supporting information can be downloaded at: https://www.mdpi.com/article/10.3390/ijms25021303/s1. References [152,153,154] are cited in the supplementary materials.
