# Supplementary material for: Ubiquitin Carboxyl-Terminal Hydrolase L1 and Its Role in Parkinson’s Disease
Source: Int J Mol Sci. 2024 Jan 21;25(2):1303. doi: 10.3390/ijms25021303 (PMC10816476; doi:10.3390/ijms25021303)
Supplement: Supplementary file 1 [file ijms-25-01303-s001.zip › ijms-2817775-supplementary.pdf]

Table S1. UCHL1-interacting proteins

#	Gene name	Protein name	Function	Localization	References
1	EGFR	Epidermal growth factor receptor	3	PM, EPR, M	1
2	NCAM1	Neural cell adhesion molecule 1	3	PM	1
3	APP	Amyloid-beta precursor protein	3	PM, EPR, M	1
4	ADRA2a	Alpha-2A adrenergic receptor	3	PM	1
5	ADRA2c	Alpha-2C adrenergic receptor	3	PM	1
6	VCL	Vinculin	2	C, PM	1
7	HSP90aa1	Heat Shock Protein 90 Alpha Family Class A Member 1	4	C, N, Mch, PM	1, 8
8	TRIM54	Tripartite motif-containing protein 54	6	C	1
9	TNIK	TRAF2 and NCK-interacting protein kinase	3	C, N	1
10	TRAF6	TNF receptor-associated factor 6	6	C, N	1
11	HNF4a	Hepatocyte nuclear factor 4-alpha	5	N	1
12	CTNND1	Catenin delta-1	3	C, N, PM, M	1
13	CTNNB1	Catenin beta-1	3	C, N, PM, M	1
14	CDKN1b	Cyclin-dependent kinase inhibitor 1B	3	C, N	1
15	UBE2L	Ubiquitin-conjugating enzyme E2L	*6*	N	1
16	TP53	TP53-binding protein 1	5	N	1
17	SNCA	Alpha-synuclein	3	C, M, N	1, 4
18	KRT17	Keratin, type I cytoskeletal 17	2	C	1
19	PTK2	Serine/threonine-protein kinase PTK2	3	C, N	1
20	PXN	Peroxisomal nicotinamide adenine dinucleotide carrier	3	P	1
21	AKT1	Potassium channel AKT1	2	PM	1

22	HTT	Huntingtin	2	C, N	1
23	ADRB2	Beta-2 adrenergic receptor	3	PM	1
24	ATG3	Ubiquitin-like-conjugating enzyme ATG3	6	C	1
25	UBB	Polyubiquitin-B	6	C, N, Mch	1
26	VHL	von Hippel-Lindau disease tumor suppressor	6	C, N, PM, EPR	1
27	CDKN2a	Tumor suppressor ARF	3	N, Mch	1
28	TRIM63	E3 ubiquitin-protein ligase TRIM63	6	C, N	1
29	USP21	Ubiquitin carboxyl-terminal hydrolase 21	6	N, C	1
30	ATG5	Autophagy protein 5	4	C	1
31	RPTOR	Regulatory-associated protein of mTOR	5	C	1
32	HSPA8	Heat shock cognate 71 kDa protein	4	C, N, PM	1
33	COPS5	COP9 signalosome complex subunit 5	3	C, N	1
34	CDK1	Cyclin-dependent kinase 1	3	N, C, Mch	1
35	SMN1/SMN2	Survival motor neuron protein	5	N, C	1
36	CDK2	Cyclin-dependent kinase 2	3	C, N	1
37	PMAIP1	Phorbol-12-myristate-13-acetate-induced protein 1	4	Mch	1
38	ADRA2b	Alpha-2B adrenergic receptor	3	PM	1
39	TUBA1a	Tubulin alpha-1A chain	2	C	1
40	LAMP2	Lysosome-associated membrane glycoprotein 2	4	PM, C, M	1
41	NEDD8	NEDD8 (Ubiquitin-like protein)	6	N	1
42	PTOV1	Prostate tumor-overexpressed gene 1 protein	5	N, C, PM, M	1
43	RANBP9	Importin-9	3	N, C	1
44	TINF2	TERF1-interacting nuclear factor 2	5	N	1

45	USP28	Ubiquitin carboxyl-terminal hydrolase 28	6	N	1
46	CBX1	Chromobox protein homolog 1	6	N	1
47	EIF1B	Eukaryotic translation initiation factor 1b	6	C	1
48	KRT4	Keratin, type II cuticular Hb4	2	C	1
49	MCC	Colorectal mutant cancer protein	3	C, N, PM	1
50	PRDX2	Peroxiredoxin-2	4	C	6
51	UBA52	Monoubiquitin	6	C, N, PM, Mch	2, 3
52	PARK2	E3 ubiquitin-protein ligase parkin	6	C, N, EPR, Mch	5
53	HSP70	Heat shock 70 kDa protein	4	C, N	7, 8
54	AMPK	5'-AMP-activated protein kinase	3	C, N	9
55	ULK1	Unc-51-like kinase 1	3	C	9
56	FUNDC1	FUN14 domain-containing protein 1	3	Mch	9
57	PKM	Pyruvate kinase PKM	1	C	9
58	MFN2	Mitofusin-2	3	Mch	10

Localization: C – cytoplasm; EPR –endoplasmic reticulum; M – membranes; PM – plasma membrane; Mch – mitochondria; N – nucleus, P – peroxisomes. Functional groups: 1. Proteins involved in energy generation and carbohydrate metabolism; 2. Proteins involved in cytoskeleton formation and exocytosis; 3. Protein involved in signal transduction and regulation of enzyme activity; 4. Antioxidant and protective proteins/enzymes; 5. Protein regulators of gene expression, cell division and differentiation; 6. Enzymes, involved in metabolism of proteins, amino acids and other nitrogenous compounds.

References

1. Jara, J.H.; Genç, B.; Cox, G.A.; Bohn, M.C.; Roos, R.P.; Macklis, J.D,; Ulupınar, E.; Özdinler, P.H. Corticospinal Motor Neurons Are Susceptible to Increased ER Stress and Display Profound Degeneration in the Absence of UCHL1 Function. *Cereb. Cortex* **2015**, 25, 259–272. doi: 10.1093/cercor/bhu318.
2. Osaka, H.; Wang, Y.L.; Takada, K.; Takizawa, S.; Setsuie, R.; Li, H.; Sato, Y.; Nishikawa K,; Sun, Y.J.; Sakurai, M.; Harada, T.; Hara, Y.; Kimura, I.; Chiba, S.; Namikawa, K.; Kiyama, H.; Noda, M.; Aoki, S.; Wada, K. Ubiquitin carboxy-terminal hydrolase L1 binds to and stabilizes monoubiquitin in neuron. *Hum. Mol. Genet.* **2003**, *12*, 1945–1958. doi: 10.1093/hmg/ddg211.

3. Meray, R.K.; Lansbury P.T. Jr. Reversible monoubiquitination regulates the Parkinson disease-associated ubiquitin hydrolase UCH-L1. *J Biol Chem*. **2007**, *282*, 10567–10575. doi: 10.1074/jbc.M611153200.
4. Liu, Y.; Fallon L.; Lashuel, H.A.; Liu, Z.; Lansbury, P.T. Jr. The UCH-L1 gene encodes two opposing enzymatic activities that affect alpha-synuclein degradation and Parkinson's disease susceptibility. *Cell* **2002**, *111*, 209–218. doi: 10.1016/s0092-8674(02)01012-7.
5. McKeon, J.E.; Sha, D.; Li, L.; Chin, L.S. Parkin-mediated K63-polyubiquitination targets ubiquitin C-terminal hydrolase L1 for degradation by the autophagy-lysosome system. Cell *Mol. Life Sci.* **2015**, *72*, 1811–1824. doi: 10.1007/s00018-014-1781-2.
6. Lee, S.P.; Park, C.M.; Kim, K.S.; Kim, E.; Jeong, M.; Shin, J.Y.; Yun, C.H.; Kim, K.; Chock, P.B.; Chae, H.Z. Structural and biochemical analyses reveal ubiquitin C-terminal hydrolase-L1 as a specific client of the peroxiredoxin II chaperone. *Arch. Biochem. Biophys*. **2018**, *640*, 61–74. doi: 10.1016/j.abb.2018.01.003.
7. Kabuta, T.; Furuta, A.; Aoki, S.; Furuta, K.; Wada, K. Aberrant interaction between Parkinson disease-associated mutant UCH-L1 and the lysosomal receptor for chaperone-mediated autophagy. *J. Biol. Chem*. **2008**, *283*, 23731–23738. doi: 10.1074/jbc.M801918200.
8. Andersson, F.I.; Werrell, E.F.; McMorran, L.; Crone, W.J.; Das, C.; Hsu, S.T.; Jackson, S.E. The effect of Parkinson's-disease-associated mutations on the deubiquitinating enzyme UCH-L1. *J. Mol. Biol*. **2011**, *407*, 261–272. doi: 10.1016/j.jmb.2010.12.029.
9. Ham, S.J.; Lee, D.; Xu, W.J.; Cho, E.; Choi, S.; Min, S.; Park, S.; Chung, J. Loss of UCHL1 rescues the defects related to Parkinson's disease by suppressing glycolysis. *Sci Adv*. **2021**, *7*:eabg4574. doi: 10.1126/sciadv.abg4574.
10. Cerqueira, F.M.; von Stockum, S.; Giacomello, M.; Goliand, I.; Kakimoto, P.; Marchesan, E.; De Stefani, D.; Kowaltowski, A.J.; Ziviani, E.; Shirihai, O.S. A new target for an old DUB: UCH-L1 regulates mitofusin-2 levels, altering mitochondrial morphology, function and calcium uptake. *Redox Biol*. **2020**, *37*, 101676. doi: 10.1016/j.redox.2020.101676.
